# UPLC/Q-TOFMS-Based Metabolomics Approach to Reveal the Protective Role of Other Herbs in An-Gong-Niu-Huang Wan Against the Hepatorenal Toxicity of Cinnabar and Realgar

**DOI:** 10.3389/fphar.2018.00618

**Published:** 2018-06-13

**Authors:** Fangbo Xia, Ao Li, Yushuang Chai, Xiao Xiao, Jianbo Wan, Peng Li, Yitao Wang

**Affiliations:** ^1^State Key Laboratory of Quality Research in Chinese Medicine, Institute of Chinese Medical Sciences, University of Macau, Macao, China; ^2^College of Pharmacy and Bioengineering, Chongqing University of Technology, Chongqing, China; ^3^Guangzhou Baiyunshan Zhongyi Pharmaceutical Co., Ltd., Guangzhou, China

**Keywords:** An-Gong-Niu-Huang Wan, cinnabar, realgar, metabonomics, detoxification, inflammation

## Abstract

An-Gong-Niu-Huang Wan (AGNH) is a well-known traditional Chinese medicine (TCM) recipe containing cinnabar (HgS) and realgar (As_2_S_2_). However, the application of AGNH is limited by the hepato- and nephrotoxicity of cinnabar and realgar. It should be noted that cinnabar and realgar in AGNH are not used alone, but rather combined with other herbs as formula to use. In this study, the protective effects and mechanisms of the other herbs in AGNH against the hepatorenal toxicity induced by cinnabar and realgar were investigated. The combination use of the other herbs in AGNH alleviated inflammatory cell infiltration and damage in the liver and kidney and restored the disturbed serum metabolic profile induced by cinnabar and realgar insults. By UPLC/Q-TOFMS combined with pattern recognition approaches, we identified 41 endogenous metabolites in the sera of mice that were related to the hepatorenal toxicity of cinnabar and realgar, 36 of which were restored to normal levels when various kinds of herbs were combined as compound recipe. These metabolites function as modulators in inflammation-associated glycerophospholipid, arachidonic acid, linoleic acid, sphingolipid, and ether lipid metabolic pathways. Notably, lysophosphatidylcholines (LysoPCs) were the most elevated among all of the metabolites detected after cinnabar and realgar treatment, while these LysoPCs did not show overt differences between the AGNH and saline control groups, which was associated with relatively unaffected or even up-regulated expression of lysophosphatidylcholine acyltransferase 1 (LPCAT1) and autotaxin (ATX). These findings indicated that other herbs in AGNH could have a protective effect against cinnabar- and realgar-induced hepatic and renal damage *via* modulating the disordered homeostasis of the glycerophospholipid, arachidonic acid, linoleic acid, ether lipid, and sphingolipid metabolism.

## Introduction

In traditional Chinese medicine (TCM), An-Gong-Niu-Huang Wan (AGNH) is widely accepted as one of the most famous formulae. The premier description of AGNH is in *Shang Jiao*, Volume One of the *Treatise on Differentiation and Treatment of Epidemic Febrile Diseases*, written by Wu Jutong in the Qing Dynasty (Guo et al., 2014). It consists of *Bovis calculus, Coptidis rhizoma, Bubali cornu, Scutellariae radix, moschus, Gardeniae fructus, margarita, Curcumae radix, Borneolum syntheticum*, cinnabar and realgar. According to previous literatures, AGNH is used to relieve specific syndromes like fever, stupor, coma, hemorrhage and to treat diseases like acute ischemic stroke, viral encephalitis, acute hemorrhagic stroke, and trauma brain injury (Lu et al., 2011a; Guo et al., 2014).

Cinnabar and realgar have been widely used in diverse formulae for 2000 years in TCM (Liu et al., 2008; Wang H. et al., 2013; Xu W. et al., 2014). In Pharmacopeia of China (2015), there are 24 recipes containing both cinnabar and realgar. Cinnabar, containing more than 96% mercuric sulfide (HgS), is mainly applied as sedative and soporific combined with other medicines. Realgar, containing more than 90% arsenic disulfide (As_2_S_2_) and other mineral arsenicals like arsenate, arsenite, and arsenic, has been applied to treat diverse diseases including boils, carbuncles, intestinal parasitosis, convulsive epilepsy, insect- and snake-bites and psoriasis in clinical practice (Liu et al., 2008). Besides, modern pharmacological studies showed arsenic exerts therapeutic effects on acute promyelocytic leukemia and other myeloid neoplasms (Chen et al., 2011; Zhang H.N. et al., 2015). Although HgS and As_2_S_2_ have been considered highly stable and less toxic *in vivo* (Lu et al., 2011b; Xu et al., 2016), the soluble mercury and arsenic existing in cinnabar and realgar preferentially bind to sulfhydryl groups present at various sites of enzymes and membranes in liver and kidney, thereby triggering oxidative stress and inflammatory process leading to further damage (Liang and Shang, 2005; Wei et al., 2008, 2009; Liang et al., 2011). Recently, a serial of clinical and experimental poisoning cases of cinnabar and realgar have been reported to associate with their overdose or long term usage (Liu et al., 2008; Zhang et al., 2017).

Due to the toxicity of cinnabar and realgar, the application and globalization of AGNH and other formulae containing them is hindered. For example, AGNH is prohibited in the United States or European markets, as mercury and arsenic content in AGNH is several folds higher than suggested by standards (Lu et al., 2011b). However, it has been proved that cinnabar and realgar are active ingredients and essential for the neuroprotection of AGNH (Lin et al., 2006; Zhu et al., 2007; Zhang F. et al., 2010). In fact, TCM is characterized by herbal combination in TCM therapy, which is known as the “seven compatibilities” including single use, mutual reinforcement, mutual assistance, mutual restraint, mutual suppression, mutual inhibition, and mutual antagonism. Among these, mutual restraint and mutual suppression are important detoxifying methods in clinical practice (Zhang G.-P. et al., 2015). For example, *Zingiberis rhizoma recens* has been applied to alleviate the toxicity of *Pinellia rhizoma* and *Arisaematis rhizoma* (Wu et al., 1999; Huang et al., 2011). When it comes to cinnabar and realgar, many studies reported the toxicity of cinnabar and realgar could be alleviated when used in combination with other herbs. Four combined herbs in Zhu-Sha-An-Shen Wan, including *Coptidis rhizoma*, uncooked *Rehmanniae radix, Angelicae sinensis radix*, and honey fried *Glycyrrhizae radix et rhizoma*, could relieved the injuries of liver and kidney induced by cinnabar (Wang H. et al., 2013). After being combined with *Scutellariae radix, Rhei radix et rhizoma, Glycyrrhizae radix et rhizoma*, and *Platycodonis radix*, acute hepatorenal toxicity of realgar in Niu-Huang-Jie-Du Pian was alleviated by synergistic detoxification effect of the above-mentioned herbs (Xu W. et al., 2014). Whether other herbal medicines in AGNH could prevent liver or kidney damage induced by cinnabar and realgar has, to our knowledge, never been investigated.

Recently, an increasing number of studies have applied metabolomics approaches in drug toxicology research (Robertson, 2005). Metabolomics is the systematic investigation of the metabolic response of living systems to any environmental stimuli, and the profiles of metabolites in biofluids, cells, and tissues can be used for biomarker discovery (Nicholson et al., 1999). In TCM research, metabolomics can integrally assess the effect or toxicity of herbs alone or TCM formula, which is in agreement with the holistic concept of TCM (Zhang A. et al., 2010; Shi et al., 2016). Currently, gas chromatography-mass spectrometry, liquid chromatography-mass spectrometry, and other hyphenated techniques are popular in metabolomics analysis of biological samples due to high sensitivity, selectivity, throughput, and depth of coverage. Moreover, metabolomics databases built based on MS and MS/MS data can offer plenty of information for the identification of metabolites (Wang et al., 2015).

To investigate the role of other herbs in the AGNH formula in attenuating the hepatorenal toxicity of cinnabar and realgar, firstly, histopathology and immunohistochemistry examinations were applied to evaluate the morphological difference between vehicle control, cinnabar and cinnabar co-administration and AGNH groups. Moreover, UPLC/Q-TOFMS-based metabolomics approach and immunoblotting were used to assess the possible detoxication mechanism.

## Materials and Methods

### Materials

An-Gong-Niu-Huang Wan (3 g per pill), cinnabar (96% HgS), and realgar (90% As_2_S_2_) were provided from Guangzhou Bai-Yun-Shan Zhong-Yi Pharmaceutical Company Ltd. (Guangzhou, China). Primary antibodies specific for the antigens were as follows: Gr-1 (BioLegend, San Diego, CA, United States); CD68 (Boster biological technology, Wuhan, China); lysophosphatidylcholine acyltransferase 1 (LPCAT1), and autotaxin (ATX; Proteintech, Chicago, IL, United States); β-actin (Bioworld Technology, Minneapolis, MN, United States).

### Animals

Adult male and female Kunming mice (20 ± 2 g) were obtained from the Experimental Animal Center of Third Military Medical University (Chongqing, China). Mice were given standard rodent chow diet and tap water *ad libitum*, and acclimatized for 1 week before all experimental procedures. All studies were carried out in full compliance with the National Institutes of Health guidelines for the Care and Use of Laboratory Animals (8th Edition, 2011). The animal care procedures and experimental protocols were reviewed and approved by the Institutional Ethics Committee of Chongqing University of Technology (Chongqing, China).

### Experimental Design

A total of 36 mice were divided randomly into three groups and each group had 12 mice. Mice received either physiological saline (control) or AGNH (2.5 g/kg, equivalent to five-fold clinical dosage) by intragastric administration once daily for consecutive 28 days. The current dose of AGNH was chosen on the basis of our pilot experiments. According to Pharmacopeia of China (2015 edition), per gram of AGNH contains equal amounts (0.056 g) of cinnabar and realgar. For comparison, a group of mice were orally administrated cinnabar (0.14 g/kg) and realgar (0.14 g/kg) per day (C+R group). On day 28, mice were euthanized with pentobarbital sodium (50 mg/kg) at 1 h after last dosing. Blood was collected by orbital bleeding under anesthesia and allowed to clot at 4°C for 1 h. After centrifugation at 1500 *g* for 10 min, the isolated sera were stored at -80°C for metabolic profile analysis. For protein analysis, the right lateral lobe of the liver and the right kidney were snap-frozen in liquid nitrogen immediately after dissection. The remaining liver and kidney tissues were fixed in 4% paraformaldehyde in 0.1 M phosphate buffer (pH 7.2), embedded in paraffin and cut serially into 4 μm sections for either histological examination or immunohistochemical analysis.

### Histopathology

After deparaffinized and stained with hematoxylin and eosin (H&E) according to standard protocols, liver and kidney tissue sections were used for histology assessment under light microscopy (Olympus Optical Co., Tokyo, Japan). Histology activity index (HAI) scoring system was used to evaluate liver injury severity (Knodell et al., 1981). Briefly, the HAI score estimated hepatocyte necrosis and inflammation in four categories, including periportal bridging necrosis (0–10), intralobular degeneradation and necrosis (0–4), portal inflammation (0–4), and fibrosis (0–4). Renal tubular injury (TI) were graded using a scale (0–5) according to the presence of tubular atrophy, dilation, and epithelial desquamation, associated with interstitial widening or intratubular cast formation, as follows: 0, normal and grade 1, changes affecting < 10%; grade 2, changes affecting 10–25%; grade 3, changes affecting 25–50%; grade 4, changes affecting 50–75%; and grade 5, changes affecting 75–100% TI involvement. A mean score corresponding to TI in each mouse was calculated based on individual values, which was determined semiquantitatively on 15 randomly chosen non-overlapping fields in the cortical and juxtamedullary regions at ×400 magnification.

### Immunohistochemistry

For immunoperoxidase staining for macrophages (CD68^+^) and neutrophils (Gr-1^+^), the deparaffinized sections were antigen retrieved by boiling for 15 min at 98°C in 10 mM citrate buffer (pH 6.0). Afterwards, hydrogen peroxide 3% (*v*/*v*) was used to eliminate endogenous peroxidase activity at room temperature for 20 min. The sections were incubated at 4°C overnight with primary antibody against CD68 or Gr-1, which was diluted to 1:100 in phosphate-buffered saline (PBS). The final incubation was carried out at 37°C for 30 min by using Polink-2 plus^®^ Polymer horseradish peroxidase (HRP) Detection System (Beijing Zhongshan Golden Bridge Biotechnology Co., Ltd., Beijing, China). The location of CD68^+^ macrophages or Gr-1^+^ neutrophils was visualized by adding the peroxidase substrate 3, 3′-diaminobenzidine (DAB) as the chromogen. Finally, slides were counterstained with hematoxylin. Negative controls were created by substituting the primary antibodies mentioned above with non-immune rat or rabbit serum. Photomicrographs of the sections (*n* = 3 per mouse) were taken using Olympus photomicrography equipment. The number of macrophage and neutrophil infiltrates was counted for each section of five randomly selected nonoverlapping high power fields (×400 magnification).

### Metabolomics Analysis

#### Sample Preparation

Serum samples were prepared following our previously published procedure with minor modification (Wang et al., 2016). Briefly, a 50 μL aliquot of serum sample was deproteinized with 150 μL of methanol and vortexed for 30 s. Then the mixed solution was centrifuged at 15,800 *g* for 15 min at 4°C, and a 200 μL aliquot of supernatant was transferred to a new sampling tube and was dried by nitrogen. Afterwards, the residue was redissolved in 50 μL water and centrifuged at 15,800 *g* for 15 min. The pooled “quality control” (QC) sample was prepared to optimize and validate the chromatographic and TOFMS conditions by mixing an aliquot of 10 μL solution from each serum sample. Each sample solution was filtered through a syringe filter (0.22 μm) before UPLC/Q-TOFMS analysis.

#### LC-MS Analysis

For each sample, an aliquot of 5 μL sample solution was separated with a Waters ACQUITY HSS T3 C18 column (100 mm × 2.1 mm, i.e., 1.7 μm) at 50°C using a Waters ACQUITY^TM^ ultra performance liquid chromatography system (Waters, Milford, MA, United States). The mobile phase, which consisted of 0.1% formic acid-water (phase A) and acetonitrile (phase B), was pumped at a flow rate of 0.4 mL/min under the following gradient program: linear gradient from 3 to 10% B (0–1 min), 10–40% B (1–3 min), 40–70% B (3–9 min), 70–80% B (9–18 min), and 80–95% B (18–20 min), isocratic 95% A for 3 min and then back to 3% B for 3 min.

The Waters SYNAPT G2-Si Q-TOF mass spectrometer with electrospray ionization (ESI) source was used to acquire serum metabolic profiles in both negative and positive ion modes. The source temperature was set at 150°C with the desolvation temperature of 500°C, the nitrogen gas flow of 600 L/h and the cone flow of 50 L/h. The capillary voltage was set to 3.0 kV in positive ion mode or 2.5 kV in negative ion mode. Centroid data was acquired across the range of 100–1000 *m*/*z*, and the scanning rate was set at 0.3 s/scan. To obtain accurate mass measurements, leucine-enkephalin solution (200 pg/mL) was continuously infused into the mass spectrometer at a low flow rate (10 μL/min).

### Western Blotting Analysis

The hepatic and renal tissue samples of about 30 mg were lysed with 150–250 μL lysis solution (Beyotime Institute of Biotechnology, Jiangsu, China) supplemented with a protease inhibitor cocktail (Roche Applied Science, Indianapolis, IN, United States). Protein concentrations in liver and kidney tissue homogenates were determined with the bicinchoninic acid (BCA) assay kit (Beyotime). Thirty micrograms of protein samples were resolved with 10% (*w*/*v*) sodium dodecyl sulphate polyacrylamide-gel electrophoresis (SDS-PAGE), and then transferred onto a polyvinylidene difluoride (PVDF) membrane (Millipore, Bedford, MA, United States). The PVDF membranes were blocked for 1 h at room temperature in Tris-buffered saline with 0.1% (*w*/*v*) Tween 20 (TBS-T) and hybridized with a 1:1000 dilution of primary antibody for LPCAT1 or ATX overnight at 4°C. Mouse monoclonal anti-β-actin antibody (1:30000 dilution) was used for protein loading control. The membrane was then incubated for 1 h at room temperature with HRP-conjugated secondary antibodies (1:3000 dilution; Bioworld Technology). Immunoreactive bands were visualized using an enhanced chemiluminescence kit (Millipore). The intensity of each band was determined using Quantity One software (Bio-Rad, Hercules, CA, United States). The variation in the intensity of bands after normalization to the β-actin was expressed as fold changes compared to that of saline-operated control.

### RNA Reverse Transcription and Quantitative Real-Time PCR (RT-qPCR)

Total RNA from hepatic and renal tissues was isolated using a RNA pure high-purity total RNA rapid extraction kit (spin-column, BioTeke, Beijing, China) according to the manufacturer’s instruction. Equal amounts of total RNA (2 μg) were reverse transcribed to cDNA using the M-MLV Reverse Transcriptase and Oligo (dT) primers (Gibco BRL, Gaithersburg, MD, United States) in a 20 μL reaction volume. Amplification of LPCAT1, ATX, and β*-actin* was performed in a 20 μL reaction mixture containing cDNA, primers (0.4 μM each of sense and antisense primers) and 2 × SYBR FAST qPCR Master Mix (KAPA Biosystems, Woburn, MA, United States). The primers of LPCAT1: 5′-AAGTGCGAGGATGAAGAGAGG (sense), 5′-CGCTGGTGTATAAGTGCTTCC (antisense); ATX: 5′-CACTACTACAGCATCATCACC (sense), 5′-CTGAGTAGGAGCCTTGATAATG (antisense); β-actin: 5′-GCTGTGCTATGTTGCTCTAG (sense), 5′-GCAACGATCACTACTGG (antisense) were synthesized by Invitrogen Life Technologies (Shanghai, China). Gene expression analysis was carried out according to the comparative Ct method (2^-ΔΔCt^), using β-actin as the housekeeping gene. Duplicate reactions were performed for each tested sample, and the average Ct was calculated for the quantification analysis.

### Statistical Analysis

The raw UPLC/Q-TOFMS data of serum samples were processed by the Progenesis QI software (Waters, Manchester, United Kingdom), a convenient data processing vehicle for omics studies. The adduct ion forms, including [M-H]^-^, [M+H]^+^, [M-H+FA]^-^ and others, were selected to achieve the fusion of precursor ions, and a data matrix containing all the metabolic features, involving retention time (t_R_), *m*/*z* and normalized peak area, was generated. After normalization, the data were processed to reduce the missing value input according to the “80% rule” (Bijlsma et al., 2006). Multivariate statistical analysis was conducted based on SIMCA-P software (version 13.0, Umetrics, Umeå, Sweden) after pareto scaling and mean-centering. The parameters including *R*^2^ and *Q*^2^ (cum) were used to assess the quality of partial least squares discriminant analysis (PLS-DA) models, and values closing to 1.0 was considered as the signal of outstanding predictive ability and fitness (Wan et al., 2013). By analyzing the metabolomics profiles of the control group and the cinnabar+realgar group with PLS-DA, potential biomarkers, mainly responsible for the discrimination between these two groups, were extracted based on variable importance in projection (VIP) values (>2.0), *S*-plot and max fold change (<2%). These putative biomarkers were identified based on accuracy molecular weight and MS/MS data in available databases including Human Metabolome Database (HMDB^1^) and METLIN.^2^

Data from 6 to 12 biological replicates were averaged for the analysis. Non-normal variables are reported as median (interquartile range [IQR]; continuous variables with normal distribution are presented as mean ± standard deviation (SD). To compare means of two and three groups of rank variables not normally distributed, statistical analysis was carried out using the nonparametric Mann–Whitney *U* test and Kruskal–Wallis test, respectively. For continuous variables, one-way analysis of variance (ANOVA) was applied to perform the comparison of different groups. If differences between the two randomized groups were significant (*P* < 0.05), multiple comparisons were performed by Fisher’s least-significant difference (LSD) *post hoc* test. SPSS Software (version 19.0, IBM, Chicago, IL, United States) was applied to analyze all data, and *P* value of less than 0.05 was considered statistically significant.

## Results

### Histopathological Evaluation

**FIGURE 1:**
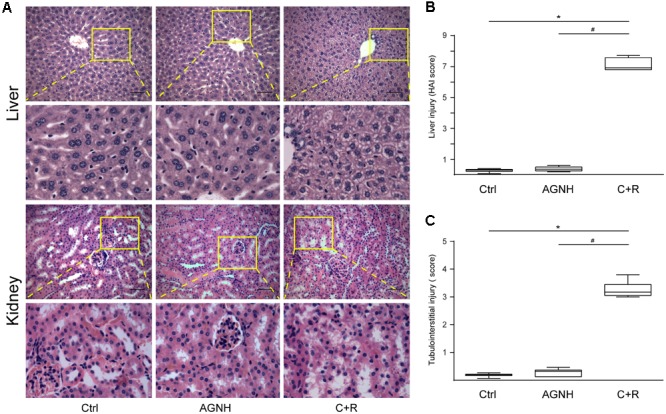
**(A)** Representative photomicrographs of liver and kidney sections stained with hematoxylin-eosin in mice from three different groups: Ctrl, saline operated; AGNH, An-Gong-Niu-Huang Wan 2.5 g/kg/d treatment for consecutive 28 days; C+R, cinnabar and reaglar 0.14 + 0.14 g/kg/d combinational treatment for consecutive 28 days. Enlarged images of the boxed regions were shown below the boxed panels. Scale bar: 50 μm. Histopathology was assessed using the **(B)** histology activity index (HA1) and **(C)** renal tubular injury (TI) scoring system in liver and kidney specimens, respectively. Horizontal bars, medians. ^∗^*P* < 0.05, saline control *versus* cinnabar and reaglar co-administration; ^#^*P* < 0.05, An-Gong-Niu-Huang Wan *versus* cinnabar and reaglar co-administration.

As shown in the **Figure 1**, histopathology results revealed that cinnabar and realgar caused significant macrovesicular steatosis and focal necrosis of hepatocytes in the centrilobular region, associated with perivenular infiltration of inflammatory cells. However, mice treated with cinnabar- and realgar-containing AGNH showed normal lobular pattern with a mild degree of fatty change and inflammatory infiltration almost comparable to the saline control. HAI score findings paralleled histological findings. The HAI score was markedly increased after cinnabar and realgar treatment. However, AGNH significantly decreased the HAI score in liver pathology.

Although no histopathological changes were found in the glomeruli between the three groups, administration of cinnabar and realgar developed TI, characterized by focal tubular atrophy, necrosis and exfoliation, existence of casts in tubules, inflammatory cell infiltration, and remarkable tubulointerstitial fibrosis. The degree of renal pathological changes was evaluated by TI scores. As shown in **Figure 1**, cinnabar and realgar caused a significant increase in TI score that was 15.85-fold higher than that in the saline control. In comparison, kidney histology in the AGNH-treated group was similar to that of the saline control, as indicated by the TI scores (merely 1.60 times that of the saline control group).

### The Effect of Cinnabar, Realgar and AGNH on the Infiltration of Inflammatory Cells Into the Liver and Kidney

**FIGURE 2:**
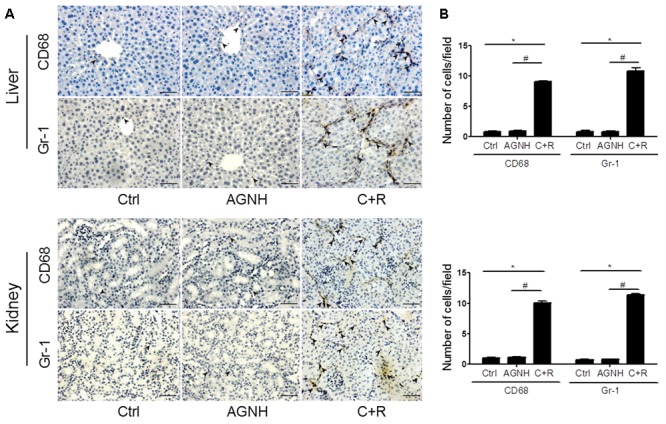
**(A)** Representative photomicrographs showing infiltrating CD68-positive macrophages or Gr-1 positive neutrophils (indicated by arrowheads) in the liver and kidney tissues of mice from three different groups: Ctrl, saline operated; AGNH, An-Gong-Niu-Huang Wan 2.5 g/kg/d treatment for consecutive 28 days; C+R, cinnabar and reaglar 0.14 + 0.14 g/kg/d combinational treatment for consecutive 28 days. Scale bar: 50 μm. **(B)** Quantification of the number of CD68-positive macrophages or Gr-1 positive neutrophils per high-power field in different groups. Each bar represents mean ± *SD* for 6 mice. ^∗^*P* < 0.05 saline control *versus* cinnabar and reaglar co-administration; ^#^*P* < 0.05, An-Gong-Niu-Huang Wan *versus* cinnabar and reaglar co-administration.

Macrophages and neutrophils are vital local effector cells in mediating liver and kidney injuries induced by cinnabar- and realgar-containing remedies. They were identified and stained with antibodies against CD68 and Gr-1 by immunohistochemistry, respectively. As shown in **Figure 2**, cinnabar+realgar markedly increased the numbers of CD68-positive macrophages and Gr-1-positive neutrophils in the liver and kidney. On the contrary, no increase in these macrophages and neutrophils after cinnabar- and realgar-containing AGNH treatment was observed when compared with the saline controls.

### UPLC/Q-TOFMS Method Validation

**FIGURE 3:**
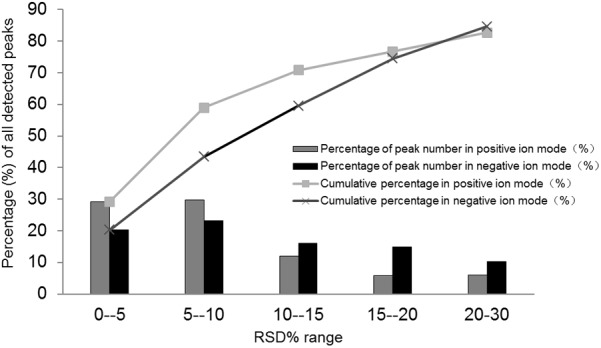
Relative standard deviation (RSD%) distribution of all metabolites in the pooled quality control (QC) samples.

In this study, to evaluate the stability and reproducibility of the UPLC/Q-TOFMS method, 10 μL from each sample were mixed to provide the QC sample. The pooled QC sample containing all metabolites was injected into the instrument three times at the beginning of sequence, and then was analyzed once every 10 samples. After alignment and normalization, the relative standard deviation (RSD%) was applied to evaluate the reproducibility of the metabolomics analysis. As shown in **Figure 3**, 82.69% of the variables among 5033 ions acquired from the QC sample had RSD% less than 30% in ESI positive ion mode, and 84.65% of variables among the 6474 ions acquired from the QC sample mode had RSD% less than 30% in ESI negative ion. All these results suggested that the analytical method could be applied in metabolomics analysis with excellent repeatability and stability.

### Metabolic Profiles in Serum Samples

**FIGURE 4:**
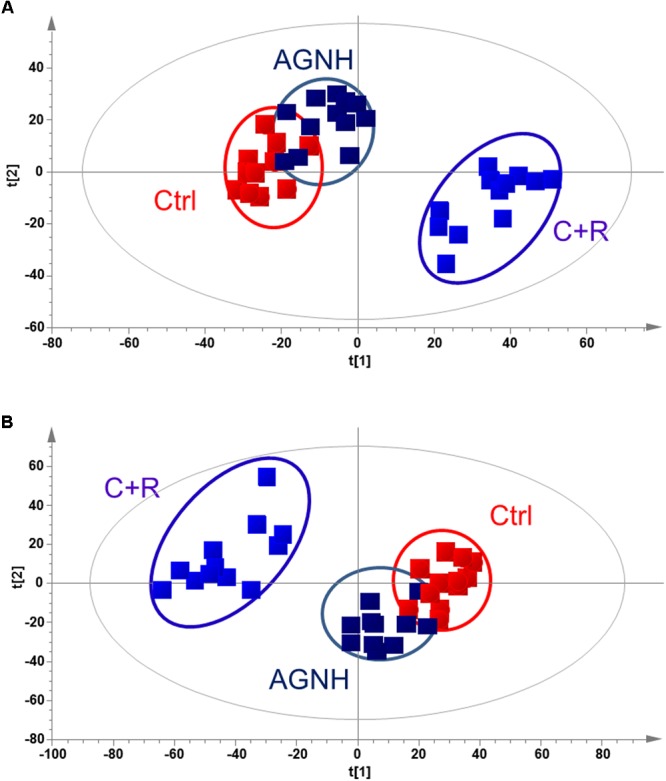
PLS-DA score plot of saline control (Ctrl), An-Gong-Niu-Huang Wan (AGNH) and cinnabar and reaglar co-administration (C+R) groups based on the serum metabolic profiles for **(A)** positive (*R*^2^*X* = 0.416, *R*^2^*Y* = 0.968, and *Q*^2^ = 0.874) or **(B)** negative (*R*^2^*X* = 0.743, *R*^2^*Y* = 0.950, and *Q*^2^ = 0.722) mode.

The metabolic profiles of control, AGNH and cinnabar+realgar groups were acquired with the validated UPLC/Q-TOFMS method. **Figure 4** showed the metabolic phenotypes of the three groups of mice using the multiple pattern recognition method. It could be observed a clear separation of metabolic phenotypes existed between the saline control and cinnabar+realgar groups in both positive and negative modes, as well as the AGNH and cinnabar+realgar groups. On the other hand, metabolic phenotype in AGNH group was similar with saline control group, suggesting that other herbs in AGNH prevented the changes of serum metabolites induced by cinnabar and realgar.

### Chemical Identification and Metabolic Pathway Analysis

**FIGURE 5:**
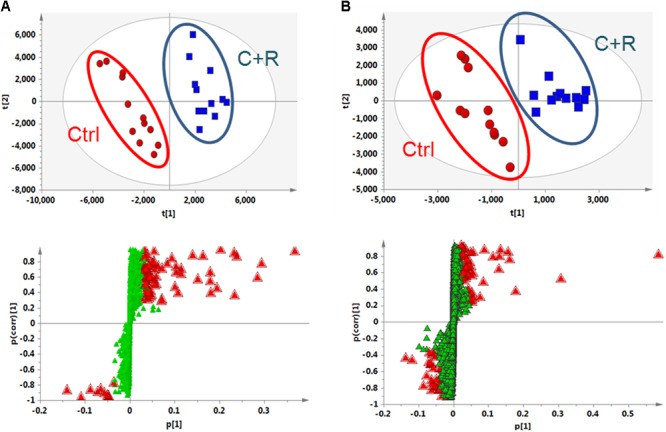
Partial least squares discriminant analysis (PLS-DA) score and *S*-plot of saline control (Ctrl) and cinnabar and reaglar co-administration (C+R) groups based on the serum metabolic profiles for **(A)** positive (*R*^2^*X* = 0.771, *R*^2^*Y* = 0.989, and *Q*^2^ = 0.932) and **(B)** negative (*R*^2^*X* = 0.507, *R*^2^*Y* = 0.944, and *Q*^2^ = 0.854) ion mode.

**FIGURE 6:**
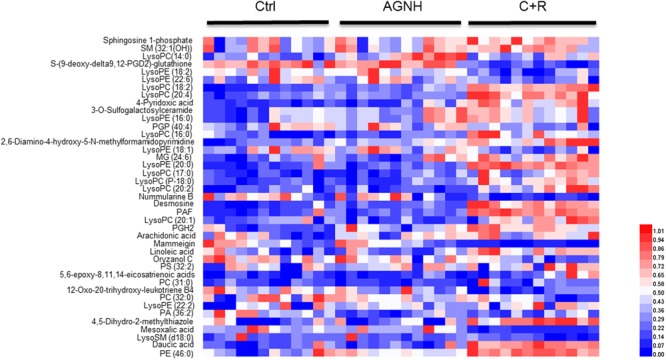
Heatmap visualizing the changes in the contents of potential biomarkers in saline control (Ctrl), An-Gong-Niu-Huang Wan (AGNH) and cinnabar and reaglar co-administration (C+R) groups. Rows: biomarkers; columns: samples. Color key indicates metabolite content value. Blue color represents low metabolite content, while red refers to high metabolite content.

**FIGURE 7:**
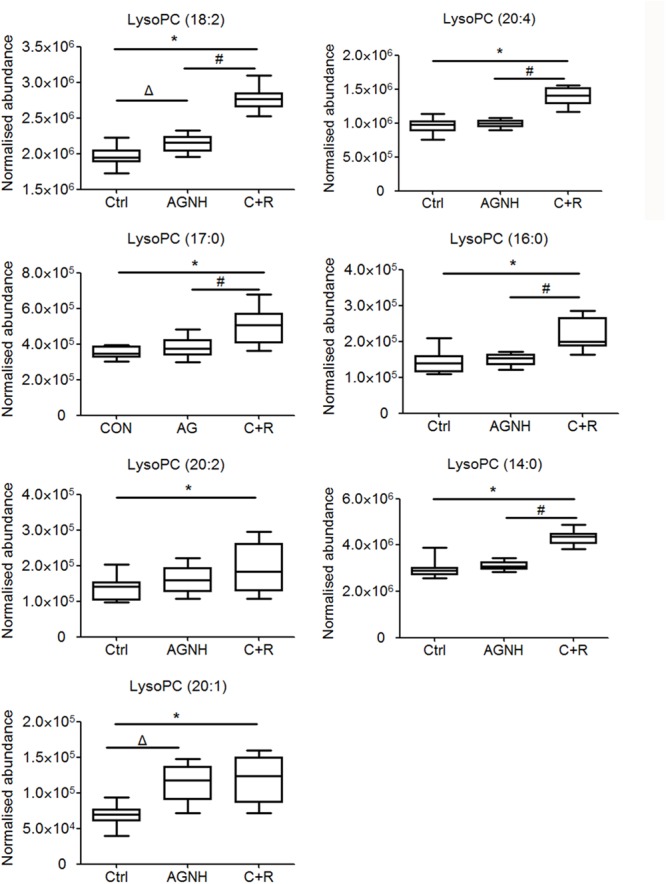
The content of lysophosphatidylcholines (LysoPCs) in sera of mice from saline control (Ctrl), An-Gong-Niu-Huang Wan (AGNH) and cinnabar and reaglar co-administration (C+R) groups. ^∗^*P* < 0.05 saline control *versus* cinnabar and reaglar co-administration; #*P* < 0.05, An-Gong-Niu-Huang Wan *versus* cinnabar and reaglar co-administration; Δ*P* < 0.05, saline control *versus* An-Gong-Niu-Huang Wan.

To clarify the underlying pathways associated with the hepatorenal toxicity of cinnabar and realgar, the metabolic profiles of control and cinnabar+realgar groups were manipulated with PLS-DA (**Figure 5**). Variables were highlighted as important for discrimination according to VIP1 and VIP2 values (>2.0), and student *t*-test was further used to filter these variables to evaluate whether these potential biomarkers were statistically significant between saline control and cinnabar+realgar groups. These filtered biomarkers were highlighted with red in the *S*-Plot (**Figure 5**). Biomarkers were tentatively identified based on molecular weight and MS/MS spectra. To identify the exact molecular formula, Progenesis QI software was used to search the biochemical data bases based on the accurate mass spectrum provided by the robust Q-TOFMS analysis platform. The accurate molecular weight and MS/MS data associated with corresponding fragment of product ion provided structural characteristics of biomarkers. Finally, 41 potential biomarkers were identified in serum samples and the results are given in Supplementary Table 1. In addition, a heatmap colored according to the relative abundances of biomarkers was constructed to visualize the difference between the two sample groups (**Figure 6**). Among all these biomarkers, the contents of lysophosphatidylcholines (LysoPCs) in cinnabar+realgar group were significantly higher than those in saline control group, but most of them including LysoPC (20:4), LysoPC (17:0), LysoPC (16:0), LysoPC (20:2), and LysoPC (14:0) were unchanged in AGNH group (**Figure 7**). Besides, a similar trend was also found for the contents of some other metabolites in serum samples (Supplementary Figure 1).

**FIGURE 8:**
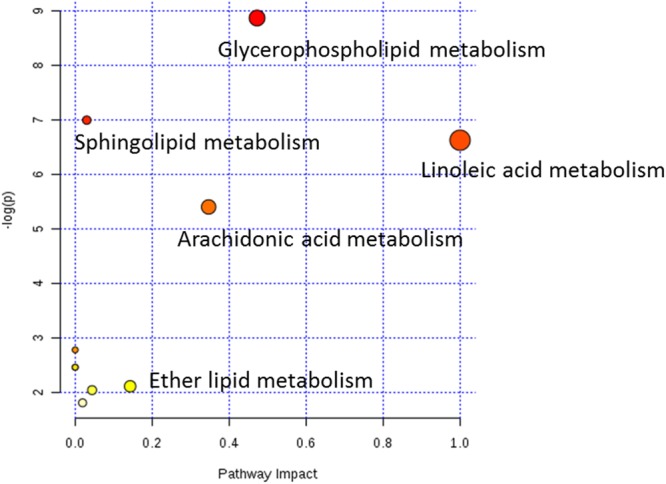
Weighted analysis of the metabolic pathways based on the potential biomarkers

To further clarify the possible metabolic pathways that were affected by other herbs in AGNH on the hepatorenal toxicity induced by cinnabar and realgar, these potential biomarkers were imported into metaboanalyst.^3^ As shown in the **Figure 8** and Supplementary Table 2, 10 metabolic pathways were highlighted. Among them, glycerophospholipid metabolism, arachidonic acid metabolism, linoleic acid metabolism, sphingolipid metabolism and ether lipid metabolism were considered as potential target pathways due to their high impact and low false discovery rate (FDR).

### Effect of AGNH and Cinnabar+Realgar on the Expression of LPCAT1 and ATX

**FIGURE 9:**
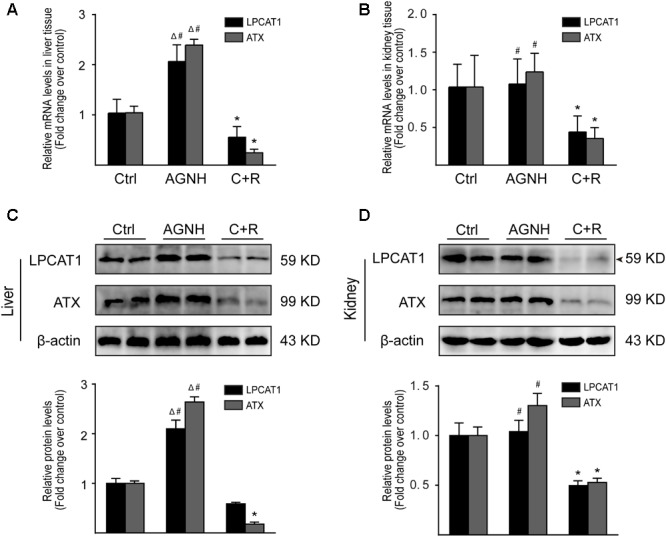
RT-qPCR analysis of gene expression was performed for LPCAT1 and ATX in the liver **(A)** and kidneys **(B)** from saline control (Ctrl), An-Gong-Niu-Huang Wan (AGNH) and cinnabar and reaglar co-administration (C+R) groups. The mRNA level of each target gene was normalized to the expression of the β-actin gene. Representative Western blotting photographs illustrating protein levels of LPCAT1, ATX and β-actin in the **(C)** liver and **(D)** kidney tissue lysates. β-Actin was used as an internal control. For quantitation of the protein levels of LPCAT1 and ATX, band intensities were converted to arbitrary densitometric units, normalized to the value of β-actin and expressed relative to the value of the same protein in vehicle-treated mice, which was defined as one-fold. Each bar represents mean ± SD for six mice. ^∗^*P* < 0.05 saline control *versus* cinnabar and reaglar co-administration; ^#^*P* < 0.05, An-Gong-Niu-Huang Wan *versus* cinnabar and reaglar co-administration; Δ*P* < 0.05, saline control *versus* An-Gong-Niu-Huang Wan.

Changes in metabolite LysoPCs are usually preceded by altered expression of two metabolic enzymes, LPCAT1 and ATX. The results obtained by RT-qPCR and Western blotting showed cinnabar+realgar resulted in a significant decrease in the mRNA and protein expression of LPCAT1 in kidney, and ATX in both liver and kidney (**Figure 9** and Supplementary Figure 2). The level of LPCAT1 protein in the liver was also slightly down-regulated without significant difference in the cinnabar+realgar group compared with the saline control group. In contrast, treatment with cinnabar- and realgar-containing AGNH showed no decrease or even an increase in the mRNA and protein levels of LPCAT1 and ATX in the liver and kidney.

## Discussion

AGNH is widely used in acute cerebrovascular conditions including stroke, encephalitis and meningitis for hundreds of years (Guo et al., 2014; Wu et al., 2016; Fu et al., 2017). However, due to the well-known hepatorenal toxicity of cinnabar and realgar in AGNH, it has been controversial for the usage of AGNH in clinical practice. In the pathogenesis of hepatorenal toxicity induced by cinnabar and realgar, inflammation has been considered as one of the most important initiating factors for hepatic and renal damage (Tacke and Zimmermann, 2014; Xu R. et al., 2014). In the present study, cinnabar and realgar co-administration indeed caused apparent toxic phenomena in liver and kidney, including significant macrovesicular steatosis and focal necrosis of hepatocytes and proximal tubules, as well as infiltrated inflammatory cells (macrophages and neutrophils). Whereas, in the liver and kidney of mice treated by cinnabar- and realgar-containing AGNH, infiltrated macrophages and neutrophils nearly disappeared and degenerated hepatocytes and renal tubular epithelial cells were attenuated. Furthermore, we have measured the serum levels of alanine aminotransferase (ALT), aspartate aminotransferase (AST), alkaline phosphatase (ALP), total bilirubin (T-BIL), albumin (ALB), creatinine, and urea nitrogen. However, neither AGNH nor C+R treatment significantly influenced the serum levels of indexes of hepatorenal toxicity, compared with those in the control group (Supplementary Table 3). Intriguingly, significant increase in the activities of the liver-specific enzymes ALT and AST in liver homogenates was observed in the C+R group compared with the saline control group. However, there were no significant differences in the levels of ALT and AST between the AGNH and saline control groups. This may be a reflection of the beginning phase of hepatocyte damage, as the liver-specific enzymes do not secrete into the blood. In addition, the change of kidney function determined through the blood tests was not consistent with renal histological lesions. This is plausible since the alteration is recovered by compensation of kidney function. Above evidence indicated the combination use of other herbs in AGNH could relieve the hepatic and renal inflammation and damage induced by cinnabar and realgar.

In fact, there have been numerous literature reporting several herbs in AGNH exert anti-inflammatory and hepatorenal protective effects. Cui et al. demonstrated that *Coptidis rhizoma* could inhibit the overproduction of inflammatory meditors in macrophages induced by lipopolysaccharide, including induced nitric oxide synthase (iNOS), nitric oxide (NO), tumor necrosis factor-α (TNF-α) and interleukin-6 (IL-6) (Cui et al., 2014). Furthermore, berberine, the main bioactive ingredient of *C. rhizoma*, could alleviate cisplatin-induced nephrotoxicity in mice by inhibiting inflammation and oxidative/nitrosative stress. Meanwhile, berberine prevented liver from ethanol-induced injury by decreasing steatosis and oxidative stress in mice (Domitrovic et al., 2013; Zhang et al., 2014). Several studies also directly demonstrated that extract of *Radix scutellariae* could effectively alleviate acute alcohol-induced liver injury and attenuate liver fibrosis induced by CCl_4_ and bile duct ligation (Nan et al., 2002; Chen et al., 2013; Dong et al., 2016). Therefore, the action mechanisms underlying ameliorative effects of other herbs in AGNH formula on cinnabar- and realgar-induced toxicity may be related with their anti-inflammatory and hepatorenal protective properties.

Metabolomics can identify potential biomarkers and multi-parametric metabolic networks by measuring and mathematically modeling the changes of metabolite levels in biological fluids and tissues. It offers an unbiased metabolism profile of entire metabolic pathways to detect the subtle dynamic changes and characterize the adverse potential when exposed to toxicants such as cinnabar and realgar. To further investigate alterations of serum metabolites after administration of cinnabar and realgar alone or in combination with other herbs as formula (AGNH), UPLC/Q-TOFMS-based metabolomics technique combining with multivariate statistical analysis methods were used to acquire and analyze the serum metabolic profile. The PLS-DA result demonstrated that there was a significant difference in the serum metabolic profiles between the control and cinnabar+realgar groups, which could be considered as a result of toxicity (**Figure 4**). However, metabolic phenotypes of AGNH group were similar with those of control group. These results consolidated the finding that other herbs in AGNH could have a protective effect against cinnabar- and realgar-induced hepatic and renal damage.

**FIGURE 10:**
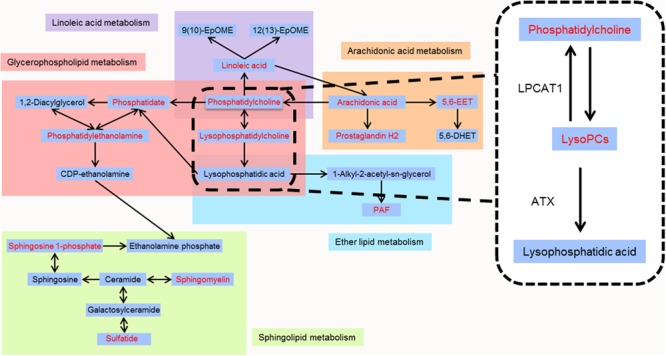
Metabolic pathways of identified metabolites. Red color represents metabolites whose content in cinnabar and reaglar co-administration group is significantly different when compared with that in saline control group.

As shown in the pathway analysis results (**Figure 10**), the glycerophospholipid metabolism can link other disturbed metabolic pathways related to the hepatoxicity and nephrotoxicity of cinnabar and realgar. LysoPC compounds are important members of glycerophospholipid metabolism pathway. The present study showed the levels of LysoPCs, including lysoPC (18:2), lysoPC (20:4), lysoPC (17:0), lysoPC (16:0), lysoPC (20:2), lysoPC (20:1), and lysoPC (14:0), were markedly elevated in the cinnabar and realgar treated mice. LysoPCs are known as not only an intermediate in glycerophospholipid biosynthesis but also a mediator in the onset of inflammation. Pro-inflammatory properties of LysoPCs may be attributed to their abilities to generate NO or reactive oxygen species (Matsubara and Hasegawa, 2005; Park et al., 2009). Recent studies have also revealed LysoPCs mediate steatosis or lipoapoptosis of hepatocytes and sepsis-associated kidney damage (Schaefer et al., 2004; Han et al., 2008; Sossdorf et al., 2013). However, there were no differences in the serum levels of lysoPC (20:4), lysoPC (17:0), lysoPC (16:0), lysoPC (20:2), and lysoPC (14:0) between the saline control and AGNH groups. Together, these findings illustrate the efficacy of other herbs in AGNH in alleviating the cinnabar- and realgar-induced disturbance of glycerophospholipid metabolism associated with hepatorenal toxicity.

By high-throughput metabolomic analysis, we found that serum LysoPC content is the most elevated among all of the biomarkers detected in mice challenged with cinnabar- and realgar-intoxication. These findings led us to postulate that excessive LysoPCs are generated as a result of unbalanced metabolic processes induced by cinnabar and realgar. In phospholipid remodeling pathway, LysoPCs are converted to phosphatidylcholines (PCs) through re-acylation by LPCAT in different tissues (Mills and Moolenaar, 2003; Uehara et al., 2016). Recent evidence has shown that in ovalbumin-induced allergic asthmatic mice, exogenous delivery of the LPCAT1 gene into the lungs attenuated airway inflammation, suggesting that LPCAT1 enzyme could remodel membrane phospholipid composition by regulating LysoPC generation during inflammation (Cheng et al., 2015). Moreover, renal inflammation and injuries caused by bacterial endotoxemia could be prevented by injecting lysophosphatidic acid (LPA), the product of LysoPC hydrolysis by the lysophospholipase D (also named ATX) (Mirzoyan et al., 2017). In our experiments, it could be observed that the expression levels of LPCAT1 and ATX proteins decreased in homogenates of liver and kidney of mice subjected to cinnabar and realgar co-administration, which contribute to an increase in serum LysoPC levels in the same animals. However, the expression of LPCAT1 and ATX is relatively unaffected (in mice kidney homogenate) or even up-regulated (in mice liver homogenate) by AGNH compared with the saline control animals. Therefore, the protective effect of other herbs in AGNH against cinnabar- and realgar-induced hepatorenal toxicity might be associated with restoration of normal metabolic pathways for LysoPC conversion.

The metabolic pathway analysis also unveiled that the identified biomarkers involved in linoleic acid and arachidonic acid metabolism were disturbed in cinnabar- and realgar-treated group. In humans, arachidonic acid, the most abundant precursor, is either catalyzed successively from linoleic acid by ∆6-desaturase, elongase and ∆5-desaturase or ingested directly as a dietary constituent (Nakamura and Nara, 2004; Guillou et al., 2010; Marchix et al., 2015). Arachidonic acid is transformed to the cyclic endoperoxide prostaglandin G2 and prostaglandin H2 by prostaglandin endoperoxide G/H synthases (PGHSs), which are colloquially called cyclooxygenases (COXs) (Funk, 2001). These chemically unstable intermediates are further metabolized to the thromboxane, prostacyclin, and prostaglandins (Ricciotti and FitzGerald, 2011). Furthermore, a portion of the arachidonic acid is metabolized rapidly by multiple cytochromes P450 to four *cis*-epoxyeicosatrienoic acids (EETs) (5,6-, 8,9-, 11,12-, and 14,15-EETs) (Zeldin, 2001). It has been shown that 5,6-EET, but not other EETs, is a substrate for COXs to form prostaglandins of the one series (Spector et al., 2004). The COXs have been extensively studied and their eicosanoid products have been shown to play an important role in the inflammatory and immune responses, as reflected by the clinical availability of the non-steroidal anti-inflammatory drugs. For example, indomethacin and other aspirin-like anti-inflammatory agents prevent arachidonic acid and 5,6-EET metabolism by inhibiting the COX metabolism of cytochromes P450 metabolites of arachidonic acid (Carroll et al., 1990). Our study showed increased levels of linoleic acid, arachidonic acid and their two downstream metabolites, PGH2 and 5,6-EET in the serum of cinnabar and realgar group compared with the saline control group (Supplementary Figure 1). In comparison, AGNH did not show overt differences from saline controls. Thus, the perturbation of arachidonic acid and its downstream metabolites might contribute to the inflammatory response in cinnabar- and realgar-induced hepatorenal injury. Other herbs in AGNH should have synergistic effects in reducing the amount of arachidonic acid-derived elicitors of inflammatory pathways.

Platelet-activating factor (PAF), the pro-inflammatory phospholipid mediator, is synthesized by *de novo* and *remodeling* pathways (Snyder et al., 1996). In the *de novo* pathway, PAF is generated from LPA by the ether lipid metabolism and related with constitutive production of PAF. The other pathway, *remodeling* pathway, also plays a vital role in stimulus-initiated PAF biosynthesis in response to external inflammatory stimuli. In the kidney, PAF is generated by both biosynthetic routes either by intrinsic mesangial cells or by infiltrating inflammatory cells and is involved in the pathogenesis of renal damage (Schlondorff and Neuwirth, 1986). Moreover, it has been shown that the concentrations of PAF in plasma and urine are significantly increased in patients with acute renal failure during septic shock, accompanying with high levels of the pro-inflammatory cytokines including IL-1β, IL-6, IL-8, and TNF-α (Mariano et al., 1999). Recently, Correa-Costa et al. have also demonstrated PAF and its G-protein coupled receptor (PAF-R) contribute to renal fibrogenesis by inducing a pro-inflammatory microenvironment, ultimately leading to renal dysfunction and progressive renal failure (Correa-Costa et al., 2014). There is also evidence that the level of PAF is obviously elevated in the cirrhotic liver of rats, with lower level of LPCAT in terms of mRNA and protein expression (Stanca et al., 2013). Here, we provide evidence that PAF levels in sera were elevated in cinnabar- and realgar-treated mice as compared to saline control mice, and meanwhile the PAF concentration of sera could be restored to normal level when treated with AGNH formula. We conclude that the inhibitory effect of other herbs in AGNH on cinnabar- and realgar-induced inflammatory cell recruitment is likely, at least in part, due to reduced PAF production.

Ceramide plays a pivotal role in sphingolipid metabolism. It serves as a precursor of most of the more complex glycosphingolipid, or as a product of the breakdown of sphingomyelin regulated by the activity of sphingomyelinase (Saddoughi et al., 2008). For example, ceramide acts as a precursor for generation of sulfatide with 3′-phosphoadenosine-5′-phosphosulfate (PAPS; “active sulfate”) as the preferred donor in sulfate transfer reactions (Vos et al., 1994). Previous results indicated sulfatide on endothelial cell serves as a native sugar ligand of L-selectin, which mediates leukocyte rolling and adhesion to endothelium at the center of inflammation. Thus, blockade of the interaction of sulfatide and L-selectin with exogenous sulfatide or sulfatide deficiency could inhibit L-selectin-dependent lymphocyte infiltration in the liver and kidney after CCl_4_ intoxication and ureteral obstruction, respectively (Ogawa et al., 2004). The catabolism of ceramide begins with the action of the enzyme ceramidase to generate sphingosine, which can be phosphorylated by sphingosine kinases 1 or 2 (SK1/2) to produce sphingosine 1-phosphate (S1P) (Nixon, 2009). Recent studies have demonstrated that in nonalcoholic steatohepatitis, an inhibitor of S1P receptor, FTY720, protected mice from liver injury by inhibiting recruitment of pro-inflammatory monocytes into the liver, providing a mechanistic link between sphingolipid signaling and inflammation (Mauer et al., 2017). Another study also showed the high fructose activated SK1/S1P signaling pathway, resulting in NF-κB activation and inflammatory responses in rat liver and BRL3A cells (Wang X. et al., 2013). Moreover, it has been demonstrated that FTY720 alleviated tubulointerstitial inflammation by suppression of the SK1/S1P pathway in a rat model of nephropathy (Xu M. et al., 2014). Consistent with previous reports, the elevation in the sera levels of sulfatide and S1P was observed after cinnabar and realgar treatment compared with the saline control group, while the levels of sulfatide and S1P in AGNH group were almost in line with those of saline control group. These findings suggest the preventive effect of other herbs in AGNH on cinnabar- and realgar-induced inflammatory cell infiltration is probably related to down-regulation of sphingolipid mediators such as sulfatide and S1P.

To the best of our current knowledge, it is the first time to study the ameliorative effects of other herbs in AGNH formula to cinnabar- and realgar-induced hepatorenal toxicity through UPLC/Q-TOFMS-based metabolomics approach combined with morphological and molecular biological methods. Our study indicated that AGNH formula was much less toxic to the liver and kidney than cinnabar and realgar in TCM clinical application. As a possible mechanism, a number of complex disturbances in the endogenous metabolites associated with cinnabar- and realgar-induced inflammation and toxicity, including LysoPCs, linoleic acid, arachidonic acid, PGH2, 5,6-EET, PAF, sulfatide, and S1P, were restored to normal in mice from AGNH, indicating that the anti-inflammatory and toxicity alleviation effects of other herbs in AGNH might be attributed to modulating the disordered homeostasis of glycerophospholipid, arachidonic acid, linoleic acid, ether lipid, and sphingolipid metabolism. The current study provides more evidence to support the safety of clinical use of cinnabar- and realgar-containing traditional Chinese medicine AGNH.

## Ethics Statement

All studies were performed in full compliance with the National Institutes of Health guidelines for the Care and Use of Laboratory Animals (8th Edition, 2011). The animal care procedures and experimental protocols were approved by the Institutional Ethics Committee of Chongqing University of Technology.

## Author Contributions

PL, JW, and YW conceived and designed the experiments. FX, XX, and AL performed the experiments. AL and FX drafted this manuscript and analyzed the data. All of the authors discussed the complete dataset to establish an integral and coherent analysis. PL and JW provided final approval of the version to be published.

## Conflict of Interest Statement

The authors declare that the research was conducted in the absence of any commercial or financial relationships that could be construed as a potential conflict of interest.

## Footnotes

1 http://www.hmdb.ca

2 http://metlin.scripps.edu

3 http://www.metaboanalyst.ca/
