# Supplementary material for: UPLC/Q-TOFMS-Based Metabolomics Approach to Reveal the Protective Role of Other Herbs in An-Gong-Niu-Huang Wan Against the Hepatorenal Toxicity of Cinnabar and Realgar
Source: Front Pharmacol. 2018 Jun 13;9:618. doi: 10.3389/fphar.2018.00618 (PMC6008407; doi:10.3389/fphar.2018.00618)
Supplement: Supplementary file 1 [file Table_1.DOCX]

# Supplementary Tables

Table 1. Identification of significantly differential metabolites in the sera of mice and their related metabolic pathways.

| RT(min) | Metabolites | Formula | *m*/*z* | MW | HMDB ID | Pathway |
| --- | --- | --- | --- | --- | --- | --- |
| 6.70 | Sphingosine-1-phosphate | C_18_H_38_NO_5_P | 379.2482n | 379.4718 | HMDB00277 | Sphingolipid metabolism |
| 6.76 | SM (32:1 (OH)) | C_37_H_73_N_2_O_7_P | 688.6482n | 688.9585 | HMDB13462 | Sphingolipid metabolism |
| 6.92 | LysoPC (14:0) | C_22_H_46_NO_7_P | 467.3009n | 467.5769 | HMDB10379 | Glycerophospholipid metabolism |
| 7.38 | S-(9-deoxy-delta9,12-PGD2)-glutathione | C_30_H_47_N_3_O_10_S | 641.2743n | 641.773 | HMDB13058 |  |
| 7.59 | LysoPE (18:2) | C_23_H_44_NO_7_P | 476.2779 | 477.5717 | HMDB11477 |  |
| 7.67 | LysoPE (22:6) | C_27_H_44_NO_7_P | 524.2788 | 525.6145 | HMDB11526 |  |
| 7.94 | LysoPC (18:2) | C_26_H_50_NO_7_P | 519.3521n | 519.6515 | HMDB10386 | Glycerophospholipid metabolism |
| 8.01 | LysoPC (20:4) | C2_8_H_50_NO_7_P | 544.3434 | 543.6729 | HMDB10395 | Glycerophospholipid metabolism |
| 8.15 | 4-Pyridoxic acid | C_8_H_9_NO_4_ | 183.0668n | 183.1614 | HMDB00017 |  |
| 8.34 | 3-O-Sulfogalactosylceramide (36:2) | C_42_H_79_NO_11_S | 805.9873n | 806.142 | HMDB12317 | Sphingolipid metabolism |
| 8.35 | LysoPE (16:0) | C_21_H_44_NO_7_P | 453.2873n | 453.5503 | HMDB11473 |  |
| 8.36 | PGP (40:4) | C_46_H_84_O_13_P_2_ | 906.5729n | 907.0989 | HMDB13513 |  |
| 8.4 | LysoPC (16:0) | C_24_H_50_NO_7_P | 496.4473*m/z* | 495.6301 | HMDB10382 | Glycerophospholipid metabolism |
| 8.47 | 2,6-Diamino-4-hydroxy-5-N-methylformamidopyrimidine | C_6_H_9_N_5_O_2_ | 184.0749 *m*/*z* | 183.168 | HMDB11657 |  |
| 8.75 | LysoPE (18:1) | C_23_H_46_NO_7_P | 479.3069n | 479.5876 | HMDB11505 |  |
| 8.8 | MG (24:6) | C_27_H_42_O_4_ | 430.4932n | 430.629 | HMDB11560 |  |
| 9.11 | LysoPE (20:0) | C_25_H_52_NO_7_P | 509.3488n | 509.6566 | HMDB11481 |  |
| 9.28 | LysoPC (17:0) | C_25_H_52_NO_7_P | 509.3496n | 509.6566 | HMDB12108 | Glycerophospholipid metabolism |
| 9.37 | LysoPC (P-18:0) | C_26_H_54_NO_6_P | 508.3772 *m*/*z* | 507.6838 | HMDB13122 | Glycerophospholipid metabolism |
| 9.4 | LysoPC (20:2) | C_28_H_54_NO_7_P | 548.3724 *m*/*z* | 547.7046 | HMDB10392 | Glycerophospholipid metabolism |
| 9.8 | Nummularine B | C_32_H_41_N_5_O_6_ | 591.4089n | 591.6978 | HMDB29334 |  |
| 10.16 | Desmosine | C_24_H_40_N_5_O_8_ | 526.3702n | 526.6031 | HMDB00572 |  |
| 10.56 | PAF | C_26_H_54_NO_7_P | 523.3184n | 523.6832 | HMDB0062195 | Ether lipid metabolism |
| 10.66 | LysoPC (20:1) | C_28_H_56_NO_7_P | 549.3799n | 549.7205 | HMDB10391 | Glycerophospholipid metabolism |
| 12.61 | PGH2 | C_20_H_32_O_5_ | 352.2196n | 352.4651 | HMDB0001381 | Arachidonic acid metabolism |
| 12.95 | Arachidonic acid | C_20_H_32_O_2_ | 303.2326 *m*/*z* | 304.4669 | HMDB01043 | Arachidonic acid metabolism;  Biosynthesis of unsaturated fatty acids |
| 12.96 | Mammeigin | C_25_H_24_O_5_ | 404.1641n | 404.4551 | HMDB30785 |  |
| 13.13 | Linoleic acid | C_18_H_32_O_2_ | 280.2410n | 280.4455 | HMDB00673 | Linoleic acid metabolism;  Biosynthesis of unsaturated fatty acids |
| 13.18 | Oryzanol C | C_41_H_60_O_4_ | 616.3965n | 616.9127 | HMDB35121 |  |
| 15.5 | PS (32:2) | C_38_H_70_NO_10_P | 731.4706n | 731.9369 | HMDB12346 |  |
| 15.51 | 5,6-Epoxy-8,11,14-eicosatrienoic acid | C_20_H_32_O_3_ | 320.1022n | 320.4663 | HMDB02190 | Arachidonic acid metabolism |
| 15.52 | PC (31:0) | C_39_H_78_NO_7_P | 703.5749 *m*/*z* | 704.0129 | HMDB11205 |  |
| 15.57 | 12-Oxo-20-trihydroxy-leukotriene B4 | C_20_H_30_O_7_ | 382.1798n | 382.448 | HMDB12553 |  |
| 19.96 | PC (32:0) | C_40_H_80_NO_8_P | 733.8192n | 734.0389 | HMDB00564 | Linoleic acid metabolism;  Glycerophospholipid metabolism;  Arachidonic acid metabolism |
| 20.05 | LysoPE (22:2) | C_27_H_52_NO_7_P | 532.5818 *m*/*z* | 533.678 | HMDB11492 |  |
| 20.07 | PA (36:2) | C_39_H_73_O_8_P | 700.5510n | 700.9659 | HMDB07861 | Glycerophospholipid metabolism;  Glycerolipid metabolism |
| 21.36 | 4,5-Dihydro-2-methylthiazole | C_4_H_7_NS | 99.9244 *m*/*z* | 101.17 | HMDB29555 |  |
| 21.4 | Mesoxalic acid | C_3_H_2_O_5_ | 117.9688n | 118.045 | HMDB31522 |  |
| 23.71 | LysoSM(d18:0) | C_23_H_51_N_2_O_5_P | 465.3031 *m*/*z* | 466.6352 | HMDB12082 |  |
| 23.74 | Daucic acid | C_7_H_8_O_7_ | 203.9616n | 204.1342 | HMDB31665 |  |
| 23.75 | PE (46:0) | C_51_H_102_NO_8_P | 886.9459 *m*/*z* | 888.3312 | HMDB09508 | Glycerophospholipid metabolism;  Glycosylphosphatidylinositol(GPI)-anchor biosynthesis |
