# Supplementary material for: UPLC/Q-TOFMS-Based Metabolomics Approach to Reveal the Protective Role of Other Herbs in An-Gong-Niu-Huang Wan Against the Hepatorenal Toxicity of Cinnabar and Realgar
Source: Front Pharmacol. 2018 Jun 13;9:618. doi: 10.3389/fphar.2018.00618 (PMC6008407; doi:10.3389/fphar.2018.00618)
Supplement: Supplementary file 2 [file Table_2.DOCX]

# Supplementary Tables

Table 2. Weighted analysis of the metabolic pathways based on the potential biomarkers related with toxicity of cinnabar and reaglar.

|  | Total | Expected | Hits | Raw p | -LOG(p) | FDR | Impact |
| --- | --- | --- | --- | --- | --- | --- | --- |
| Glycerophospholipid metabolism | 30 | 0.2964 | 4 | 0.000141 | 8.8639 | 0.011595 | 0.47253 |
| Sphingolipid metabolism | 21 | 0.20748 | 3 | 0.000921 | 6.9904 | 0.036356 | 0.03008 |
| Linoleic acid metabolism | 6 | 0.05928 | 2 | 0.00133 | 6.6225 | 0.036356 | 1 |
| Arachidonic acid metabolism | 36 | 0.35568 | 3 | 0.004526 | 5.398 | 0.092775 | 0.34677 |
| Biosynthesis of unsaturated fatty acids | 42 | 0.41496 | 2 | 0.062309 | 2.7757 | 1 | 0 |
| alpha-Linolenic acid metabolism | 9 | 0.08892 | 1 | 0.085719 | 2.4567 | 1 | 0 |
| Vitamin B6 metabolism | 9 | 0.08892 | 1 | 0.085719 | 2.4567 | 1 | 0 |
| Ether lipid metabolism | 13 | 0.12844 | 1 | 0.12158 | 2.1072 | 1 | 0.14286 |
| Glycosylphosphatidylinositol(GPI)-anchor biosynthesis | 14 | 0.13832 | 1 | 0.13034 | 2.0376 | 1 | 0.0439 |
| Glycerolipid metabolism | 18 | 0.17784 | 1 | 0.16457 | 1.8044 | 1 | 0.0192 |
