# Supplementary material for: UPLC/Q-TOFMS-Based Metabolomics Approach to Reveal the Protective Role of Other Herbs in An-Gong-Niu-Huang Wan Against the Hepatorenal Toxicity of Cinnabar and Realgar
Source: Front Pharmacol. 2018 Jun 13;9:618. doi: 10.3389/fphar.2018.00618 (PMC6008407; doi:10.3389/fphar.2018.00618)
Supplement: Supplementary file 3 [file Table_3.DOCX]

# Supplementary Tables

Table 3. The results of biochemical analysis in mice of liver and kidney injuries (*n* = 12).

| Index | Ctrl | AGNH | C+R |
| --- | --- | --- | --- |
| ALT (serum; U/L) | 39.2 ± 13.4 | 35.7 ± 18.7 | 37.6 ± 12.6 |
| AST (serum; U/L) | 60.4 ± 19.6 | 55.3 ± 12.4 | 62.0 ± 13.3 |
| ALP (serum; KA. U/100mL) | 20.8 ± 6.2 | 25.5 ± 12.8 | 23.2 ± 8.1 |
| T-BIL (serum; μmol/L) | 3.6 ± 1.4 | 3.5 ±0.9 | 3.6 ± 1.2 |
| ALB (serum; g/L) | 7.7 ±1.8 | 6.6 ± 1.8 | 8.2 ± 2.8 |
| Creatinine (serum; *μ*mol/L) | 23.3 ±17.7 | 30.6 ± 15.1 | 34.0 ± 15.4 |
| Urea nitrogen (serum; mmol/L)  ALT (liver; U/g protein) | 3.0 ± 0.5  2.2 ± 0.3 | 2.6 ± 0.5  2.1 ± 0.2^#^ | 2.6 ± 0.6  2.5 ± 0.2^*^ |
| AST (liver; U/g protein) | 1.5 ± 0.2 | 1.6 ± 0.4^#^ | 1.9 ± 0.2^*^ |

**P* < 0.05, *versus* saline control (Ctrl); #*P* < 0.05, *versus* cinnabar and reaglar co-administration (C+R).
