# Supplementary material for: UPLC/Q-TOFMS-Based Metabolomics Approach to Reveal the Protective Role of Other Herbs in An-Gong-Niu-Huang Wan Against the Hepatorenal Toxicity of Cinnabar and Realgar
Source: Front Pharmacol. 2018 Jun 13;9:618. doi: 10.3389/fphar.2018.00618 (PMC6008407; doi:10.3389/fphar.2018.00618)
Supplement: Supplementary file 4 [file Image_1.PDF]

Supplementary Material

UPLC/Q-TOFMS-based Metabolomics Approach to Understand the Hepatorenal Toxicity Alleviation Effect of Other Herbs in An-Gong-Niu-Huang Wan to Realgar and Cinnabar

Fangbo Xia[1†], Ao Li[1,2†], Yushuang Chai[3], Xiao Xiao[2], Jianbo Wan[1*], Peng Li[1*], Yitao Wang[1]

[1]State Key Laboratory of Quality Research in Chinese Medicine, Institute of Chinese Medical Sciences, University of Macau, Macao 999078, China

[2]College of Pharmacy and Bioengineering, Chongqing University of Technology, Chongqing 400054, China

[3]Guangzhou Baiyunshan Zhongyi Pharmaceutical Co., Ltd, Guangzhou 510530, China

† These authors contributed equally to this work.

* Correspondence:

Jianbo Wan, jbwan@umac.mo; Peng Li, pli1978@hotmail.com

1 Supplementary Figure

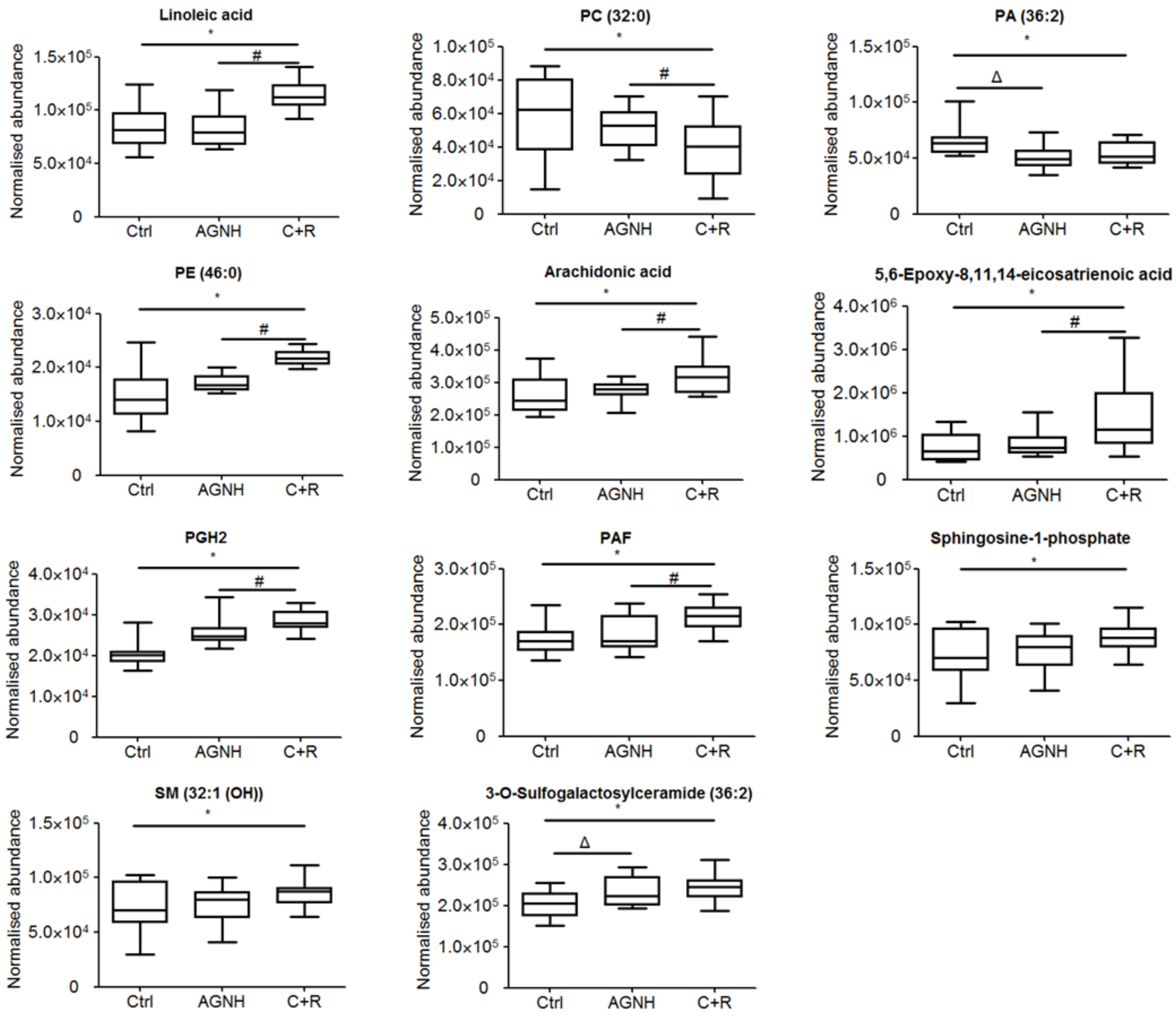

Supplementary Figure 1. The content of some metabolites in sera of mice from saline control (Ctrl), An-Gong-Niu-Huang Wan (AGNH) and cinnabar and reaglar co-administration (C+R) groups. *P < 0.05 saline control *versus* cinnabar and reaglar co-administration; #P < 0.05, An-Gong-Niu-Huang Wan *versus* cinnabar and reaglar co-administration; ΔP < 0.05, saline control *versus* An-Gong-Niu-Huang Wan.
