# Supplementary material for: UPLC/Q-TOFMS-Based Metabolomics Approach to Reveal the Protective Role of Other Herbs in An-Gong-Niu-Huang Wan Against the Hepatorenal Toxicity of Cinnabar and Realgar
Source: Front Pharmacol. 2018 Jun 13;9:618. doi: 10.3389/fphar.2018.00618 (PMC6008407; doi:10.3389/fphar.2018.00618)
Supplement: Supplementary file 5 [file Image_2.PDF]

1 Supplementary Figure

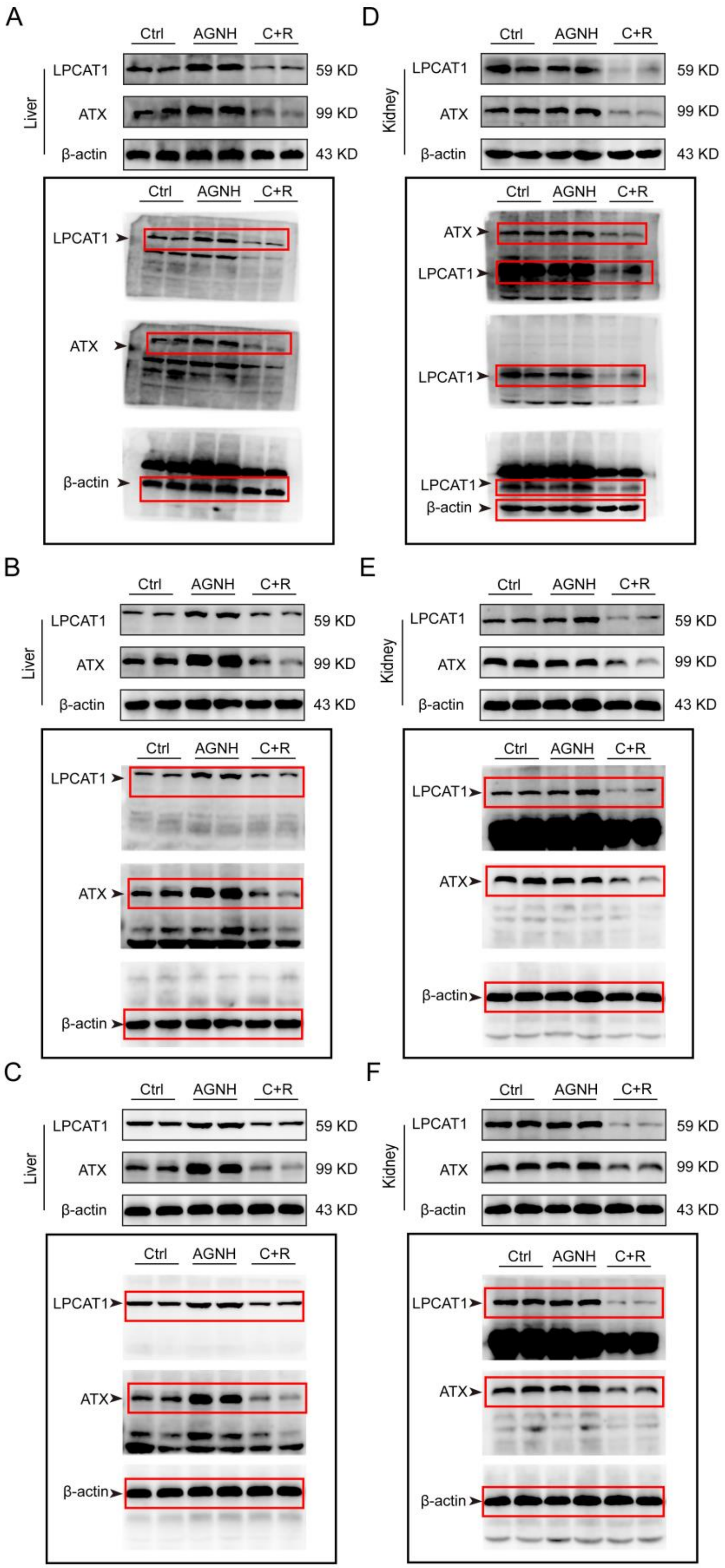
A
Ctrl
AGNH
C+R
Liver
LPCAT1
59 KD
ATX
99 KD
β-actin
43 KD
B
C
D
E
F
Kidney

Supplementary Figure 2. Additional Western blotting images illustrating protein levels of LPCAT1, ATX and *β*-actin in the (A, B) liver and (C, D) kidney tissue lysates from saline control (Ctrl), An-Gong-Niu-Huang Wan (AGNH) and cinnabar and reaglar co-administration (C+R) groups. Red lines show the cropping locations.
